# When lockdown policies amplify social inequalities in COVID-19 infections: evidence from a cross-sectional population-based survey in France

**DOI:** 10.1186/s12889-021-10521-5

**Published:** 2021-04-12

**Authors:** Nathalie Bajos, Florence Jusot, Ariane Pailhé, Alexis Spire, Claude Martin, Laurence Meyer, Nathalie Lydié, Jeanna-Eve Franck, Marie Zins, Fabrice Carrat, for the SAPRIS study group, Nathalie Bajos, Fabrice Carrat, Pierre-Yves Ancel, Marie-Aline Charles, Florence Jusot, Claude Martin, Laurence Meyer, Alexandra Rouquette, Ariane Pailhé, Gianluca Severi, Alexis Spire, Mathilde Touvier, Marie Zins

**Affiliations:** 1 Institut de Recherche Interdisciplinaire sur les enjeux Sociaux - Sciences sociales, politique, santé, IRIS (UMR 8156 CNRS - EHESS - U997 INSERM), 5 cours des humanités, 93322 Aubervilliers, France; 2 Paris Dauphine University, Paris, France; 3 National Institute for Demographic Studies, Paris, France; 4 French National Centre for Scientific Research, Paris, France; 5 University of Paris Sud, Paris, France; 6 Santé publique France, Paris, France; 7 Sorbonne University, Paris, France

**Keywords:** Social inequalities, Lockdown, COVID-19, General population, Risk factors

## Abstract

**Background:**

Significant differences in COVID-19 incidence by gender, class and race/ethnicity are recorded in many countries in the world. Lockdown measures, shown to be effective in reducing the number of new cases, may not have been effective in the same way for all, failing to protect the most vulnerable populations. This survey aims to assess social inequalities in the trends in COVID-19 infections following lockdown.

**Methods:**

A cross-sectional survey conducted among the general population in France in April 2020, during COVID-19 lockdown.

Ten thousand one hundred one participants aged 18–64, from a national cohort who lived in the three metropolitan French regions most affected by the first wave of COVID-19.

The main outcome was occurrence of possible COVID-19 symptoms, defined as the occurrence of sudden onset of cough, fever, dyspnea, ageusia and/or anosmia, that lasted more than 3 days in the 15 days before the survey. We used multinomial regression models to identify social and health factors related to possible COVID-19 before and during the lockdown.

**Results:**

In all, 1304 (13.0%; 95% CI: 12.0–14.0%) reported cases of possible COVID-19. The effect of lockdown on the occurrence of possible COVID-19 was different across social hierarchies. The most privileged class individuals saw a significant decline in possible COVID-19 infections between the period prior to lockdown and during the lockdown (from 8.8 to 4.3%, *P* = 0.0001) while the decline was less pronounced among working class individuals (6.9% before lockdown and 5.5% during lockdown, *P* = 0.03). This differential effect of lockdown remained significant after adjusting for other factors including history of chronic disease. The odds of being infected during lockdown as opposed to the prior period increased by 57% among working class individuals (OR = 1.57; 95% CI: 1.00–2.48). The same was true for those engaged in in-person professional activities during lockdown (OR = 1.53; 95% CI: 1.03–2.29).

**Conclusions:**

Lockdown was associated with social inequalities in the decline in COVID-19 infections, calling for the adoption of preventive policies to account for living and working conditions. Such adoptions are critical to reduce social inequalities related to COVID-19, as working-class individuals also have the highest COVID-19 related mortality, due to higher prevalence of comorbidities.

**Supplementary Information:**

The online version contains supplementary material available at 10.1186/s12889-021-10521-5.

## Introduction

Given the pre-existing social inequalities in health within societies [1] and the significant differences in COVID-19 mortality by gender, class and origin recorded in countries such as France [2, 3], the United Kingdom [4], the USA [5] and other countries around the world [6], several studies address issues of social inequalities related to COVID-19 [7–11]. However, to our knowledge, no study has investigated potential social inequalities in the effects of lockdown policies, widely implemented around the globe.

Our hypothesis is that lockdown measures, shown to be effective in reducing the number of new cases [12], have not been effective in the same way for all, failing to protect the most vulnerable populations. While more privileged social classes may have had greater exposure to the virus prior to lockdown, due to more frequent social interactions in public spaces (e.g. bars, restaurants) and travelling, they may have better adapted to lockdown measures, through telework, while working classes may have benefited less from lockdown conditions, due to their professional obligations as essential workers and their living conditions in overcrowded housing.

Our objective was to study the differential effect of lockdown measures on possible COVID-19 infections according to social class in France, one of the most affected countries in Europe by the first wave of COVID-19.

## Methods

### Study design and participants

The SAPRIS (*SAnté, Pratiques, Relations et Inégalités Sociales en population générale pendant la crise COVID-19*) survey was set-up mid-March 2020, with the general aim of understanding the main epidemiological, social and behavioural challenges of the SARS-CoV2 epidemic in France [13]. It relies on a *consortium* of five prospective population-based cohort. The analysis presented here is based on data from one of the three adult cohorts, the *Constances* cohort, which is the only cohort to have accurate data on professional status and preventive measures in the workplace. *Constances* is a generalist cohort made up of a national sample of 215,000 adults aged 18 to 69 at inclusion and recruited from 2012 onwards [14].

All cohort members of *Constances* who had regular access to the internet (*n* = 66,848) were invited to complete the SAPRIS questionnaire online. 69.0% participated in the survey (46,107). To best highlight the impact of the lockdown on possible COVID-19 symptoms, we chose to center this analysis on individuals (18–64 years) who have already been employed, living in one of the three metropolitan French regions most affected by the first wave of COVID-19 i.e. Grand Est, Ile-de-France (Paris Region) and Hauts-de-France. Ten thousand one hundred one participants met this criteria and were included in the analysis.

Data were collected in accordance with the Declaration of Helsinki. Ethical approval and written informed consent was obtained from each participant to be included in the initial cohort in 2015. In accordance with French law, this nested survey, based on an ad hoc COVID-19 questionnaire administered in 2020 to cohort participants, did not require any specific additional written consent from the participant. This nested COVID-19 survey has been approved by the Inserm ethics review committee (approval no. 20–672 of March 30, 2020).

### Data collection

Data collected online from April 6th to May 5th, 2020 solicited information on socio-demographic characteristics, household size and composition, employment characteristics, daily life conditions, childcare arrangements, alcohol and tobacco use, sexual life, comorbidities, health care utilization and treatments. The questionnaire also addressed COVID-19 related topics including preventive behaviors (gel, mask, social distancing) for individuals and in the workplace, risk perceptions and COVID-19 related beliefs as well as a detailed description of COVID-19 symptoms over the last 2 weeks.

Symptoms were reported if they were unusual and occurred at least once in the past 2 weeks (“*In the last two weeks, including today, have you had any of the following symptoms that you do not usually have: fever/cough/difficulty breathing/twitching of taste or smell ...*”). The duration of symptoms were graded on a scale of one to five (less than 1 day, 1 to 3 days, 4 to 7 days, 8 to 14 days, > 14 days). Finally, the total time (in days) between the onset of the first symptom and the date of the survey was reported.

### Measures

Our main outcome was a three-category measure, distinguishing 1) No suspicion of Covid-19 infection, 2) probable infection before the lockdown and 3) probable infection during the lockdown. We used the following criteria defined by the European Centre for Disease Prevention to identify “possible COVID-19 infection: at least cough or fever or dyspnea or sudden onset of ageusia, dysgeusia or anosmia occurring during the at-risk period” [15]. We added an additional criterion of duration, including symptoms lasting more than 3 days to add additional specificity to our definition [16].

As underlined by McAloon and colleagues, the choice of which parameter values are adopted depends on how the information is used, the associated risks and the perceived consequences of decisions to be taken [17]. In France, the incidence of the first wave was estimated to be highest around the 19th of March - during the first week of the lockdown - and rapidly decreased right after [15]. Therefore, the probability of having been infected by the COVID-19 was higher before rather than during the lockdown. The period of incubation was chosen to be 7 days (the 75th percentile of the incubation period [18]) in order to class the individuals which have had their symptom(s) on the first week of the lockdown in the “likely infection prior lockdown” group, according to the higher probability of having been infected before rather than during the lockdown.

The likely period of infection (LPC) was identified as a function of *i)* the duration between the onset of the first symptoms and the date of the survey (DFS), *ii* the duration of incubation (DI) of 7 days [16] and *iii)* the date of survey (DS). LPS was defined as follows:

$$ LPC= DS-\left( DFS+ DI\right) $$

Based on this information probable infection before the lockdown included LPC before March 17th while infection during the lockdown included LPC on or after March 17th.

Participants’ social position was defined according to 3 criteria: current professional status (Inactive, retired or unemployed before the beginning of the pandemic/ employed but stopped working since the beginning of the pandemic / Full-time teleworking / Full-time or part-time in-person professional activities)*,* social class and financial situation as perceived by respondents (comfortable/no problems/difficult). Social class was based on current or previous occupation, and distinguished health professions with specific exposure to the virus. The following 5 categories were constructed: Health professionals (doctors, nurses, caregivers), Upper class (senior managers), High middle class (intermediate professions), Low middle class (employees and skilled workers with a diploma of higher or equal to 2 years university degree), working class (unskilled employees and workers with a diploma lower than a 2years university degree).

### Statistical methods

We used inverse probability weighting to correct for selection and non-participation biases. The *Constances* cohort includes a randomly selected representative sample of the French adult population and is affiliated to the General Health Insurance Fund (the source population, that is, approximately 85% of the total French population [14]. By January 2020, 204,973 participants had been recruited in the *Constances* cohort, 66,848 were regular internet users and were invited to the survey and 46,107 did participate. Our sampling weights had the objectives to adjust for selection (66,848 among 204,973) and participation/non-response (46,107 among 66,848). Sampling weights were estimated by two different logistic regression models, with selection or participation as the response variables and with participant characteristics collected before the survey from all cohort participants (Region, Rural/Urban living, age, sex, socio-professional category, educational level, number of people living in the household, BMI, smoking, perceived health, comorbidities) as covariates. The weight adjusted on selection and participation (range: 2.75–7.32) was obtained from the product of weights estimated from the selection (range: 2.08–8.65) and participation (range: 1.15–2.49) regression models.

Then calibration of these weights was performed by generalized raking [19] in relation to the marginal totals of the age-class, gender and socio-professional-category distributions in the 2020 source population at the regional level. A final trimming excluded all observations with weights exceeding the 99th percentile.

Since the information on the number of rooms in the housing unit was only asked in a second survey in June 2020, this information was missing for the 22% of the sample who didn’t complete the second questionnaire. We imputed this data using predictions obtained by logistic regression.

Sociodemographic area characteristics (size of the agglomeration and region), number of individuals living in the household per room, educational level, nationality (French or not French), professional status, smoking, body mass index, health status (chronic diseases) were described and compared using Chi2 tests between the three possible COVID-19 infection groups. We then conducted a multinomial logistic regression to compare the risk of infection before (reference category) and during the lockdown according to social class, with successive and additional adjustments for other socio-demographic and health factors. The final model presents the variables that allow us to test our hypotheses on the effect of living conditions: housing, social class and professional status.

All analyses were performed using R software (R 3.6.1). A *P*-value < 0.05 was considered statistically significant. All percentages are weighted to account for the complex sampling design and post stratification.

Multivariable analyses were performed on unweighted data.

### Role of the funding source

The funders had no role in the design, analysis, interpretation or writing. All the authors had full access to all the data and NB and FC had final responsibility for the decision to submit for publication.

## Results

Table 1 describes the sociodemographic and health characteristics distribution of study participants and the frequency of possible COVID-19, according to the probable date of infection.

**Table 1:** Participants characteristics and associated proportion of possible COVID-19 by period

	Weighted distribution	likely infection	*P*-Value*	likely infection prior the lockdown	likely infection during the lockdown	*P*-Value**
***Age***			**0.003**			**0.002**
18–34	25.2 (1759)	13.8 (232)		8.3 (145)	5.5 (87)	
35–44	29.7 (3028)	14.8 (425)		8.3 (267)	6.6 (158)	
45–54	25.8 (2816)	12.6 (378)		8.7 (268)	3.9 (110	
55–64	19.3 (2498)	9.7 (269)		6.2 (193)	3.5 (76)	
***Sex***			0.947			0.783
Female	52.4 (5164)	13 (663)		8.2 (442)	4.8 (221)	
Male	47.6 (4937)	13 (641)		7.8 (431)	5.2 (210)	
***Nationality***			0.437			0.709
French	96.1 (9703)	13.1 (1263)		8.1 (842)	5.1 (421)	
Not french	3.9 (348)	11.3 (37)		7.3 (28)	4 (9)	
***Social Class***			0.965			0.234
Upper class	32.7 (5541)	13 (718)		8.8 (500)	4.3 (218)	
Upper middle class	20.9 (2397)	12.9 (303)		8.5 (205)	4.4 (98)	
Lower middle class	18.1 (894)	13.4 (114)		8 (72)	5.4 (42)	
Working class	21.8 (838)	12.4 (108)		6.9 (66)	5.5 (42)	
Health professional	6.5 (431)	13.9 (61)		6.2 (30)	7.7 (31)	
***Professional status***			**0.018**			**0.002**
Unemployed at the time of lockdown’s onset	24.5 (2178)	10.6 (241)		7.2 (177)	3.5 (64)	
Employed and stopped working since COVID	18.0 (1185)	15.7 (181)		10.3 (116)	5.4 (65)	
Full time teleworking	38.8 (5469)	12.9 (719)		8.1 (492)	4.7 (227)	
in-person professional activities	18.7 (1269)	13.7 (163)		6.6 (88)	7.1 (75)	
***Overcrowding***			0.077			0.183
less or equal than one pers./room	94.4 (9557)	12.8 (1210)		7.9 (816)	4.9 (394)	
more than one pers./room	5.6 (544)	16.3 (94)		9.5 (57)	6.7 (37)	
***Financial resources***			**0.046**			0.077
At ease	29.5 (4082)	12.8 (493)		8.3 (346)	4.5 (147)	
No particular problem	46.5 (4369)	12.1 (563)		7.5 (382)	4.5 (181)	
Difficult	24.1 (1581)	15.2 (238)		8.7 (138)	6.6 (100)	
***Region***			**0.02**			**0.006**
Ile-de-France	38.7 (5195)	14.4 (714)		8.9 (482)	5.5 (232)	
Grand Est	30.6 (2854)	13.4 (368)		9.1 (257)	4.3 (111)	
Hauts-de-France	30.7 (2052)	10.9 (222)		5.8 (134)	5.1 (88)	
***Agglomeration size***			0.21			0.268
Rural area	6.9 (488)	15.2 (67)		10.1 (47)	5.1 (20)	
< 50,000	4.3 (335)	12 (44)		7.3 (30)	4.7 (14)	
50–200,000	8.2 (536)	11.6 (67)		7.5 (49)	4.1 (18)	
200–500,000	17.1 (1972)	10.9 (229)		7.6 (157)	3.3 (72)	
500,000–1,000,000	24.8 (1564)	12.2 (180)		6.5 (106)	5.6 (74)	
> 1,000,000	38.8 (5200)	14.4 (715)		8.9 (482)	5.5 (233)	
***Chronic disease***			**<0.0001**			**<0.0001**
None	77.7 (7923)	12.9 (1001)		7.8 (664)	5 (337)	
Hypertension	4.7 (455)	10.2 (54)		4.7 (33)	5.5 (21)	
Asthma or other respiratory diseases	2.6 (246)	28.8 (61)		19.2 (44)	9.6 (17)	
Diabetes, cancer, heart disease, heart disease, immune diseases, liver, kidney, immunity,	2.4 (257)	12.6 (34)		10 (24)	2.6 (10)	
Others	12.7 (1220)	11.8 (154)		7.5 (108)	4.3 (46)	
***Active smoking***			0.807			0.856
Yes, daily	11.7 (819)	12.9 (93)		7.3 (57)	5.6 (36)	
Yes, sometimes (less than once a day)	4.6 (348)	11.3 (43)		6.2 (26)	5.1 (17)	
No	83.8 (8727)	13.1 (1136)		8.1 (770)	4.9 (366)	
***Obesity***			0.811			0.448
BMI < 30	85.9 (8889)	12.9 (1133)		7.8 (758)	5.1 (375)	
BMI ≥ 30	14.1 (868)	13.3 (124)		9.1 (84)	4.1 (40)	
***Individual preventive measures*** *(mask, gel, social distancing)* ***during outings in the last 7 days.***			**<0.0001**			**<0.0001**
All 3	27 (2797)	16.2 (413)		9.7 (277)	6.5 (136)	
At least one	63 (6355)	11.3 (750)		7 (497)	4.4 (253)	
None	10 (949)	14.7 (141)		10 (99)	4.6 (42)	
***All***	100 **(10,101)**	13.0 (1304)		8.0 (873)	5.0 (431)	

*Chi2 test likely infection /no infection

**Chi2 test between no infection/prior/during the lockdown

The sample was equally divided between men (47.6%) and women (52.4%) and the mean age was 43.50 years (95%CI: 43.17–43.83). More than a third (38.8%) of the sample lived in cities with more than 1,000,000 inhabitants while a minority (6.9%) lived in rural areas. About a third of the sample (32.7%) were considered upper class while 21.8% were working class. 15.3% of the sample had in person professional activities during the lockdown period.

Altogether, 13.0% (95% CI: 12.0–14.0%) of participants reported symptoms compatible with possible cases of COVID-19 (*n* = 1335) in the 2 weeks preceding the survey.

Residents from the Paris region (Ile-de-France) (*P* = 0.02), participants facing financial difficulties (*P* = 0.046) and those who reported chronic conditions (asthma or respiratory pathologies specifically) (*P* = 0.0001) were more likely to report possible COVID-19 while older participants (*P* = 0.003), and those who did not work before lockdown (*P* = 0.033) were less likely to report those symptoms. Reporting possible COVID-19 was unrelated to social class.

**Fig. 1:**
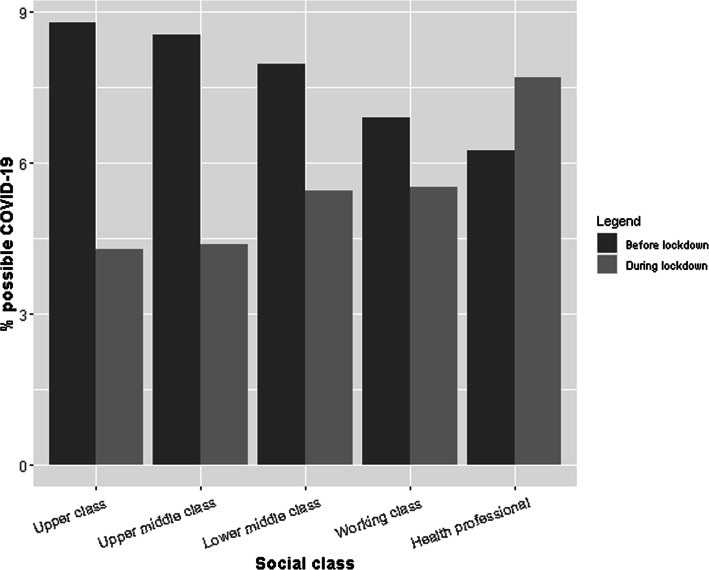
Percentage of individuals likely to be infected before or during lockdown by social class

While the percentage of participants reporting possible COVID-19 infection during lockdown was lower than participants reporting possible COVID-19 infection before lockdown (5.0% versus 8.0%, *P* = 0.001), this decrease was uneven across social groups. As shown in Fig. 1, the decline was most pronounced among privileged classes (from 8.8% before lockdown to 4.3% during lockdown, *P* = 0.001) while the decline was least pronounced among the working class (from 6.9% before lockdown to 5.5% during lockdown, *P* = 0.03).

In addition, those living in housings with less than one room *per* person were slightly more likely to report a possible case of COVID-19 than others (16.3% versus 12.8%, *P* = 0.08), with no difference between before and during lockdown.

The multivariable analyses presented in Table 2 indicated that the odds of no infection relative to probable infection prior to lockdown was unrelated to social class but depended on the region of residence, with increased odds among residents from the Hauts-de-France region relative to those residing in the Paris region (Ile de France) (OR = 1.39; 95% CI: 1.13–1.71, *P* = 0.002).

**Table 2:** Factors associated with possible COVID-19: adjusted OR (95% CI) Multinomial regression results Reference group: probable infection prior to the lockdown. OR adjusted for all the variables presented in the table

	no symptoms/likely infection prior the lockdown	*P*-Value	likely infection during the lockdown/likely infection prior the lockdown	*P*-Value
***Age***				
18–34	1		1	
35–44	0.95 (0.77–1.17)	0.622	1.00 (0.72–1.38)	0.99
45–54	0.87 (0.71–1.07)	0.198	0.69 (0.49–0.97)	**0.035**
55–64	1.09 (0.86–1.37)	0.475	0.73 (0.49–1.07)	0.101
***Sex***				
Female	1		1	
Male	1.00 (0.86–1.15)	0.95	1.03 (0.82–1.30)	0.809
***Social Class***				
Upper class	1		1	
Upper middle class	0.98 (0.82–1.18)	0.861	1.09 (0.80–1.48)	0.594
Lower middle class	1.15 (0.87–1.50)	0.322	1.28 (0.84–1.97)	0.251
Working class	1.11 (0.83–1.49)	0.467	1.57 (1.00–2.48)	**0.051**
Health professional	1.05 (0.69–1.61)	0.82	1.66 (0.91–3.04)	0.098
***Professional status***				
Unemployed at the time of lockdown’s onset	1		1	
Employed and stopped working since COVID	1.03 (0.85–1.25)	0.77	0.79 (0.56–1.12)	0.181
Full time teleworking	0.84 (0.67–1.05)	0.126	1.11 (0.78–1.58)	0.568
in-person professional activities	1.25 (0.96–1.62)	0.104	1.53 (1.03–2.29)	**0.037**
***Overcrowding housing***	0.80 (0.60–1.06)	0.121	1.24 (0.80–1.90)	0.338
***Region***				
Ile-de-France	1		1	
Grand Est	0.99 (0.83–1.18)	0.924	0.84 (0.63–1.13)	0.258
Hauts-de-France	1.39 (1.13–1.71)	**0.002**	1.26 (0.91–1.74)	0.168

111 (1%) participants excluded from the multivariate model due to missing values including 41 with possible COVID-19. Chronic disease, obesity, smoking and individual preventive measures are not presented in the final model since the odds ratio for the social class remained of the same magnitude (Supplementary Table 1)

Regarding the risk of infection during lockdown relative to the risk of infection before lockdown, it was higher among participants who had in-person professional activities compared to those who worked remotely (OR = 1.53; 95% CI: 1.03–2.29, *P* = 0.037). This risk was also increased among working class compared to upper class participants (OR = 1.57; 95% CI: 1.00–2.48, *P* = 0.051). It is worth noting that the odds-ratio for working class was 1.53 (95% CI: 0.96–2.42, *P* = 0.071) when adjusting for smoking and it was 1.49 (95%CI: 0.93–2.40, *P* = 0.096) when adjusting for history of chronic disease and obesity. Finally, this odds-ratio was reduced to 1.43 (95% CI: 0.90–2.28, *P* = 0.128) when adjusting for perceived financial situation.

## Discussion

To our knowledge, SAPRIS is the largest general population-based COVID-19 study in Europe that simultaneously collects detailed data on symptoms and social characteristics to investigate the impact of lockdown on possible COVID-19 infections.

Analyses by time period, corresponding to whether individuals may have been infected before or during lockdown, show differential trends by social class that were masked in an overall analysis. The issue of temporality is essential because confinement measures affected individuals differently according to their housing and working conditions [5, 20]. Individuals at the top of the social hierarchy saw a greater decline in COVID-19 symptoms after the lockdown than those from the working class. In fact, working-class individuals were more likely than those in the upper class to have been infected during lockdown rather than before.

Our results show that this overexposure during lockdown was partly a result of their health status and lifestyle (smoking and history of chronic disease and obesity) since the OR for the working class slightly decreased after adjustment for these variables. Likewise, it was also partly an effect of their economic precariousness since the OR of the working class decreased when it was adjusted for this variable. The latter result is consistent with economists’ work that has recently established that “the prevalence of the epidemic was higher in the poorer communes in France [21]. We also found that living in housing with less than one room *per* person tended to be linked to the risk of having been infected. Finally, our results do not reflect a lower propensity of the working class to adopt individual prevention measures.

One can think that the overexposure to the virus of the working class during lockdown may reflect, at least in part, the fact that more individuals belonging to this class live in neighborhoods with high population density (Bajos et al. [22]). Such an effect is not completely captured by the size of the agglomeration. For example, the density in some neighborhoods in the Paris suburbs, where excess mortality by COVID-19 is particularly high, is higher than that observed in larger cities (https://dashboard.covid19.data.gouv.fr/vue-d-ensemble?location=FRA). The association between population density and SARS-CoV-2 transmission rates is well established [23, 24]. Residents of these high-density cities might have had more difficulty avoiding close contacts while shopping, on the street or on public transport. In any case, the data suggest that working class individuals were less protected by the lockdown measures than the more privileged categories.

Our results also show that continuing to work outdoors is associated with an increased risk of infection during lockdown. This could result from an increased number of contacts during public transport to work and also with colleagues. Finally, the increase in possible COVID-19 among health care workers despite the diffusion of masks probably reflects the persistence of contact with patients highly contagious.

This analysis has several limitations. First, the sample is socially diverse but is not fully representative of the French population as it only represents three regions in France and respondents from the Constances cohort who have internet connectivity are not representative of all residents in France. In particular, the study fails to capture particularly vulnerable groups such as undocumented migrants and homeless people. The few studies that have been carried out on vulnerable populations clearly show the importance of social and economic resources in dealing with the epidemic [8, 11].

While the study provides information on social class based on education and employment, it doesn’t capture other forms of social disadvantage including race and ethnicity that are shown to increase the risk of COVID-19 infection in many settings and the risk COVID-19 related mortality in France [3] and other countries [25–27].

Additionally, it should be noted that our analyses are based on reported symptoms rather than on biologically tested cases, thus excluding asymptomatic individuals. However, the shortage of tests did not permit the use of testing in this study conducted in the early stages of the pandemic, especially before lockdown, as the use of RT-PCR testing was limited to patients with severe symptoms. The share of participants reporting symptoms compatible with possible cases of COVID-19 (13%) may appears high with respect to the infection spread in France during the same period of reference. Nevertheless, these are symptoms/illness without virological confirmation. Furthermore, our study was performed in the three regions which were the most affected by the first wave of COVID-19 in France (with seroprevalence estimates in the range of 7 to 10–11% [16]). In other representative studies, possible COVID-19 symptoms largely exceeded seroprevalence. For example, in the Pollan’s study [25], the seroprevalence was 5% and the rate of participants reporting possible COVID-19 symptoms was 14%.

Another limitation relates to the fact that some people may have had COVID-19 symptoms prior to the 15 days of the survey and are not counted in our possible COVID-19 cases. Since the socio-demographic structure of the respondents is stable during the study period (not shown), it is reasonable to think that the de facto exclusion of these situations does not affect results on association of possible Covid19 with social class.

In addition, although symptom reporting may risk being socially differentiated, it is reasonable to assume that any social reporting bias does not vary during the month of the survey.

In any case, from a prevention perspective, it is important to characterise the most exposed social groups and to try to uncover the social logics that favour this exposure, particularly those referring to living conditions [28, 29].

In conclusion, we showed that the effect of a lockdown policy designed and applied without taking into account social characteristics can contribute to increasing social inequalities in exposure to the risk of contracting the virus, as was rightly pointed out recently by several authors [30–32]. In this sense, the biomedical approach to prevention, which promotes preventive measures based on clinical knowledge without taking into account the socially differentiated effects of living conditions shows its limits, as was the case in the fight against previous epidemics [9, 33]. Our results call for the implementation of future preventive policies that tackle these social inequalities. Such implementation is critical to reduce social inequalities related to COVID-19, as working-class individuals also have the highest COVID-19 related mortality, due to higher prevalence of comorbidities [11].

## Supplementary Information

**Additional file 1: Supplementary Table 1.** Factors associated with possible COVID-19: adjusted OR (95% CI) Multinomial regression results. Additional adjustments on chronic disease, obesity, smoking and individual preventive measures.
