# Supplementary material for: When lockdown policies amplify social inequalities in COVID-19 infections: evidence from a cross-sectional population-based survey in France
Source: BMC Public Health. 2021 Apr 12;21:705. doi: 10.1186/s12889-021-10521-5 (PMC8040364; doi:10.1186/s12889-021-10521-5)
Supplement: Supplementary file 1 — Additional file 1: Supplementary Table 1. Factors associated with possible COVID-19: adjusted OR (95% CI) Multinomial regression results. Additional adjustments on chronic disease, obesity, smoking and individual preventive measures. [file 12889_2021_10521_MOESM1_ESM.docx]

*When lockdown policies amplify social inequalities in COVID-19 infections. Evidence from a cross-sectional population-based survey in France”*

Nathalie Bajos, PhD^1*^, Prof Florence Jusot, PhD^2^, Ariane Pailhé, PhD^3^, Alexis Spire, PhD^4^, Claude Martin, PhD^4^, Prof Laurence Meyer, PhD^5^, Nathalie Lydié, PhD^6^, Jeanna-Eve Franck, PhD^1^, Prof Marie Zins, PhD^7^, Prof Fabrice Carrat, PhD^7^; for the SAPRIS study group*

Supplementary Table 1: Factors associated with possible COVID-19: adjusted OR (95% CI)

Multinomial regression results. Additional adjustments on chronic disease, obesity, smoking and individual preventive measures.

*Reference group:* probable infection *prior to the lockdown*

*OR adjusted for all the variables presented in the table*

|  | no symptoms/likely infection prior the lockdown | P-Value | likely infection  during the lockdown/likely infection prior the lockdown | P-Value |
| --- | --- | --- | --- | --- |
| ***Age*** |  |  |  |  |
| 18-34 | 1 |  | 1 |  |
| 35-44 | 0.95 (0.76-1.17) | 0.609 | 1.00 (0.71-1.39) | 0.985 |
| 45-54 | 0.89 (0.72-1.11) | 0.313 | 0.69 (0.48-0.98) | **0.039** |
| 55-64 | 1.13 (0.89-1.45) | 0.323 | 0.70 (0.46-1.05) | 0.085 |
| ***Sex*** |  |  |  |  |
| Female | 1 |  | 1 |  |
| Male | 0.96 (0.83-1.11) | 0.541 | 1.03 (0.81-1.31) | 0.807 |
| ***Social Class*** |  |  |  |  |
| Upper class | 1 |  | 1 |  |
| Upper middle class | 0.98 (0.81-1.19) | 0.846 | 1.06 (0.77-1.46) | 0.731 |
| Lower middle class | 1.14 (0.86-1.50) | 0.359 | 1.31 (0.85-2.02) | 0.226 |
| Working class | 1.13 (0.84-1.54) | 0.420 | 1.45 (0.90-2.34) | 0.130 |
| Health professional | 1.08 (0.69-1.68) | 0.742 | 1.52 (0.81-2.86) | 0.190 |
| ***Professional status*** |  |  |  |  |
| Unemployed at the time of lockdown’s onset | 1 |  | 1 |  |
| Employed and stopped working since COVID | 1.08 (0.88-1.32) | 0.459 | 0.89 (0.62-1.27) | 0.510 |
| Full time teleworking | 0.85 (0.68-1.07) | 0.168 | 1.13 (0.79-1.64) | 0.500 |
| In-person professional activities | 1.26 (0.95-1.65) | 0.103 | 1.57 (1.04-2.38) | **0.033** |
| ***Overcrowding housing*** | 0.79 (0.59-1.05) | 0.105 | 1.21 (0.78-1.88) | 0.401 |
| ***Region*** |  |  |  |  |
| Ile-de-France | 1 |  | 1 |  |
| Grand Est | 1.00 (0.83-1.19) | 0.969 | 0.85 (0.63-1.15) | 0.283 |
| Hauts-de-France | 1.49 (1.20-1.87) | **0.000** | 1.38 (0.97-1.95) | 0.070 |
| ***Chronic disease*** |  |  |  |  |
| None | 1 |  | 1 |  |
| Hypertension | 1.11 (0.76-1.62) | 0.590 | 1.40 (0.77-2.54) | 0.271 |
| Asthma or other respiratory diseases | 0.43 (0.30-0.61) | **0.000** | 0.82 (0.46-1.48) | 0.520 |
| Diabetes, cancer, heart disease, heart disease, immune diseases, liver, kidney, immunity, | 0.85 (0.55-1.31) | 0.452 | 0.95 (0.45-2.04) | 0.904 |
| Others | 0.92 (0.74-1.15) | 0.487 | 0.79 (0.53-1.17) | 0.245 |
| ***Active smoking*** |  |  |  |  |
| Yes, daily | 1 |  | 1 |  |
| Yes, sometimes (less than once a day) | 0.84 (0.51-1.37) | 0.474 | 0.81 (0.38-1.71) | 0.573 |
| No | 0.71 (0.53-0.94) | **0.019** | 0.67 (0.43-1.04) | 0.075 |
| ***Obesity*** |  |  |  |  |
| BMI<30 | 1 |  | 1 |  |
| BMI≥30 | 0.82 (0.64-1.05) | 0.111 | 0.98 (0.65-1.46) | 0.903 |
| ***Individual preventive measures*** *(mask, gel, social distancing)* ***during outings in the last 7 days.*** |  |  |  |  |
| All 3 | 0.96 (0.74-1.26) | 0.790 | 1.06 (0.67-1.67) | 0.798 |
| At least one | 1.26 (0.98-1.63) | 0.074 | 1.11 (0.72-1.7) | 0.650 |
| None | 1 |  | 1 |  |
